# Domestic Animal Hosts Strongly Influence Human-Feeding Rates of the Chagas Disease Vector *Triatoma infestans* in Argentina

**DOI:** 10.1371/journal.pntd.0002894

**Published:** 2014-05-22

**Authors:** Ricardo E. Gürtler, María C. Cecere, Gonzalo M. Vázquez-Prokopec, Leonardo A. Ceballos, Juan M. Gurevitz, María del Pilar Fernández, Uriel Kitron, Joel E. Cohen

**Affiliations:** 1 Laboratory of Eco-Epidemiology, Department of Ecology, Genetics and Evolution, Universidad de Buenos Aires-IEGEBA (CONICET-UBA), Buenos Aires, Argentina; 2 Department of Environmental Studies, Emory University, Atlanta, Georgia, United States of America; 3 Laboratory of Populations, Rockefeller and Columbia Universities, New York, New York, United States of America; Universidad Autónoma de Yucatán, Mexico

## Abstract

**Background:**

The host species composition in a household and their relative availability affect the host-feeding choices of blood-sucking insects and parasite transmission risks. We investigated four hypotheses regarding factors that affect blood-feeding rates, proportion of human-fed bugs (human blood index), and daily human-feeding rates of *Triatoma infestans*, the main vector of Chagas disease.

**Methods:**

A cross-sectional survey collected triatomines in human sleeping quarters (domiciles) of 49 of 270 rural houses in northwestern Argentina. We developed an improved way of estimating the human-feeding rate of domestic *T. infestans* populations. We fitted generalized linear mixed-effects models to a global model with six explanatory variables (chicken blood index, dog blood index, bug stage, numbers of human residents, bug abundance, and maximum temperature during the night preceding bug catch) and three response variables (daily blood-feeding rate, human blood index, and daily human-feeding rate). Coefficients were estimated via multimodel inference with model averaging.

**Findings:**

Median blood-feeding intervals per late-stage bug were 4.1 days, with large variations among households. The main bloodmeal sources were humans (68%), chickens (22%), and dogs (9%). Blood-feeding rates decreased with increases in the chicken blood index. Both the human blood index and daily human-feeding rate decreased substantially with increasing proportions of chicken- or dog-fed bugs, or the presence of chickens indoors. Improved calculations estimated the mean daily human-feeding rate per late-stage bug at 0.231 (95% confidence interval, 0.157–0.305).

**Conclusions and Significance:**

Based on the changing availability of chickens in domiciles during spring-summer and the much larger infectivity of dogs compared with humans, we infer that the net effects of chickens in the presence of transmission-competent hosts may be more adequately described by zoopotentiation than by zooprophylaxis. Domestic animals in domiciles profoundly affect the host-feeding choices, human-vector contact rates and parasite transmission predicted by a model based on these estimates.

## Author Summary

The major vectors of Chagas disease are species of triatomine bugs that have adapted to human sleeping quarters and may feed on domestic animals and humans. There is a striking lack of information on the blood-feeding rates of Triatominae in field conditions, and factors modifying the fraction of bugs that feed on humans have rarely been investigated. Here we tested whether the spring fraction of bugs' feeding contacts with humans would decrease when dogs and chickens are available in human sleeping quarters in a well-defined endemic rural area in northwestern Argentina. On average, late-stage bugs fed every four days in spring and the majority fed on humans. The results demonstrate that both the percentage of human-fed bugs and the daily rate of feeding on humans decreased substantially with increasing proportions of chicken- or dog-fed bugs in the house, or the presence of chickens indoors. Combined with earlier findings on the seasonal dynamics of bug abundance, host-feeding patterns and the relative infectivity of domestic hosts, the net effects of the presence of chickens and dogs in human sleeping quarters is predicted to increase the transmission of *T. cruzi* in summer when chickens move outdoors.

## Introduction

The host species composition in a habitat, relative host abundance and proximity affect the host-feeding choices of hematophagous insect vectors and associated parasite transmission risks [1]. Host defensive behavior in response to attacks and the density of blood-sucking insects also modify host-feeding patterns in triatomine bugs, vector of Chagas disease [2], and in blackflies, vectors of river blindness [3]. The estimation of host choices and blood-feeding rates, two key parameters used to estimate vectorial capacity in mathematical models of vector-borne diseases [4], is fraught with difficulties [5].

Zooprophylaxis is the use of wild or domestic animals, which are not the reservoir hosts of a given disease, to divert the blood-seeking mosquito vectors from the human hosts of that disease [6]. Its use for malaria prevention dates back to the early twentieth century [7], and was strongly advocated as a supplementary control strategy in the 1980s [8]. Empirical evidence showed conflicting results in various parts of the world. Cattle sometimes increased, and sometimes decreased, the proportion of *Anopheles* mosquitoes that fed on humans [9,10 and references therein]. Although the malaria vector *Anopheles arabiensis* took a high proportion of meals from cattle, the vector population fed sufficiently on humans to transmit malaria and therefore classical zooprophylaxis may not have a significant impact [11]. Mathematical models predicted that under certain conditions of strong host-feeding preference for the protective host, the diversion of mosquito vectors would reduce malaria transmission [12]. Computer simulations predicted that reducing the mosquitoes' access to humans would have a much greater effect on the transmission of malaria than for Japanese encephalitis (which has animal reservoir hosts), where the most critical factor would be the proximity of the reservoir hosts to vector breeding sites [13]. For sand fly vectors of visceral leishmaniasis, the effects of cattle on vector abundance and transmission were also mixed [14]. For tick-borne Lyme disease in northeastern USA, the relative proportion of white-footed mice and Virginia opossums was predicted to affect transmission risks [15], but the net outcome may depend on the presence of other more or less competent hosts [7].

Both the concept of zooprophylaxis and its more recent relative, the "dilution effect" [16], propose that the probability of vectors feeding on transmission-competent hosts would decline with increasing numbers of non-competent hosts. Non-competent hosts would also reduce the abundance of infected vectors and transmission risks under certain, not all, circumstances [7], [17]. The opposite of zooprophylaxis is zoopotentiation [13]: exacerbated parasite transmission by wild or domestic animals that are not the reservoir hosts of a given disease.

*Triatoma infestans* –the most important vector of the kinetoplastid protozoan *Trypanosoma cruzi* that causes Chagas disease in humans and mammalian hosts– is well adapted to thrive in human sleeping quarters and surrounding habitats occupied by domestic animals [18], [19]. This trait, combined with epidemiological significance, susceptibility to modern pyrethroids and political momentum, justified targeting *T. infestans* for elimination in the southern cone countries of South America since 1991 [19]. Large-scale insecticide spraying campaigns suppressed this species from extensive areas in Brazil, Chile and Uruguay where transmission of human *T. cruzi* infection through *T. infestans* was interrupted [20]. Elsewhere in the southern cone countries and more particularly in the Gran Chaco ecoregion extending over Argentina, Bolivia and Paraguay, insecticide control campaigns have reduced but not eliminated transmission of human *T. cruzi* infection [21]. Although the available information is sparse and disease control status heterogeneous, the magnitude of the problem is reflected in the high seroprevalence of *T. cruzi* in pregnant women examined mostly at public health services from 1999 to 2004, which ranged from 5 to 20% in the most affected provinces in the Argentinean Chaco [22]. Regarding vector-borne transmission, annual incidence rates of human infection rose to 4.3-4.6% in some highly infested regions of the Argentinean and Bolivian Chaco [23], [24].

The notion of zooprophylaxis has been debated in Chagas disease [2,25 and references therein]. In human sleeping quarters, *T. infestans* mainly feeds on humans, dogs, chickens and cats throughout its distribution range [2], [26], [27]. Chickens and other birds are not susceptible to *T. cruzi* and avian blood exerts no effects on an established bug infection (though it supports the development and reproduction of bugs), whereas dogs and cats are key reservoir hosts of *T. cruzi* [21], [25]. In households from three rural villages in northwestern Argentina, the proportion of domestic *T. infestans* that blood-fed on humans (i.e., human blood index) decreased substantially with the increasing presence of chickens or dogs in human sleeping quarters and with increasing domestic bug abundance during spring-summer [2]. “Domestic bug” refers to bugs captured in the set of contiguous human sleeping quarters and rooms that share a continuous roof structure (i.e., domestic areas or domiciles). The presence of chickens nesting in domestic areas correlated positively with increased bug abundance and with an increasing proportion of chicken-fed bugs [28]; reduced the proportion of *T. cruzi*-infected domestic *T. infestans*, and increased the abundance of infected vectors [25]. Moreover, the adjusted odds of human infection with *T. cruzi* increased with the household number of infected dogs or cats and the presence of chickens indoors [29]. An experimental study corroborated that the additional presence of chickens relative to infected guinea pigs reduced bug infection prevalence [30]. A mathematical model of the household transmission of *T. cruzi* was consistent with this empirical evidence and predicted that the fraction of spring bugs' feeding contacts with humans would decrease with more dogs and chickens in spring because both animal hosts would divert vector feeding contacts [31]. This prediction remained untested in the absence of an appropriate method to estimate blood-feeding rates and field data.

There is a striking lack of information on the blood-feeding rates of Triatominae in field conditions. Feeding rates of domestic *T. infestans* and *Rhodnius prolixus* were first estimated indirectly by the distributions of bug weight and length in 2 and 14 houses, respectively [32], [33]. A more widely applicable method estimated daily feeding rates by measuring the temperature-adjusted occurrence of transparent (clear) urine assessed shortly after capture [34]. Using this method, domestic populations of *T. infestans* were estimated to blood-feed every three days over the spring-summer period [35], and those from chicken coops fed more often than bugs from other peridomestic habitats in spring [36], [37]. The product of the human blood index and daily feeding rate is not a daily rate because of the undefined period of time during which the bugs may have fed on a given host species, and because the human blood index cannot distinguish whether the bugs fed once or several times on humans [24]. Therefore, in this study we developed a different way of calculating human-feeding rates that provides more informative estimates and a measure of its variability among houses in a population-based survey.

We also collected additional data to estimate the daily blood-feeding rates, host choices, and rates of human-bug feeding contacts per person of domestic *T. infestans* from the same study region as our previous investigations, although in the context of lower domestic infestations. Factors modifying the fraction of bugs that feed on humans have rarely been investigated. Using generalized linear mixed-effects models (GLMM) and a multimodel inference approach with model averaging, here we test the following hypotheses related to domestic *T. infestans*: 1) Blood-feeding rates should increase with host availability (i.e., humans, chickens, dogs) and ambient temperature [38]; 2) The human blood index and human-feeding rate should decrease with more chicken or dog blood meals and the occurrence of indoor-nesting chickens and dogs (i.e., greater host diversity and abundance) [2]; 3) The human blood index and human-feeding rate should decrease with increasing bug abundance per unit of sampling effort because of increasing host irritation with increasing bites per host [2], and 4) The predicted number of human-bug feeding contacts per person-day should decrease with the indoor presence of chickens and dogs in spring [31]. Testing these hypotheses is important for risk assessment and a better understanding and control of domestic populations of Triatominae. Our current results support and extend previous findings and the main predictions of the mathematical model.

## Materials and Methods

### Study area

Field work was carried out in October-November 2003 in eight neighboring rural communities with 270 houses in Figueroa Department (27° 23′S, 63° 29′W), Province of Santiago del Estero, Argentina, described elsewhere [39]. The study area was selected for an insecticide trial in 2003–2005 because it had high infestation with *T. infestans* and recent occurrence of human acute cases of *T. cruzi* despite a previous community-wide residual spraying with pyrethroid insecticides conducted in 2000. Most houses were made of adobe walls and thatched roofs, with one or two adjacent bedrooms and a front verandah 5–10 m wide, and had multiple peridomestic structures for housing domestic animals. A weather station (Weather Monitor II, Davis, Baltimore, MD) located 50 km from the study area measured temperature, relative humidity, wind speed and direction, barometric pressure, and rainfall at 15 min intervals.

### Study design

A cross-sectional survey of house infestation was conducted before control interventions in October-November 2003 [39]. Each of the 270 houses was visited and georeferenced. The location and type of building material of each structure were recorded in a sketch map and in a form. All houses positive for *T. infestans* during the first week of the survey (October 20–27, 2003) were considered eligible for blood-feeding studies. Time constraints for processing the bugs within 8 hours of capture (needed to estimate blood-feeding frequency) dictated that insects from domestic areas at 49 different house compounds were processed (i.e., 52% of houses with domestic areas found infested). These 49 sites were all of the sites that satisfied the time constraints, and were not a sample of a larger number of possible sites.

Householders were asked about the number of human occupants aged 15 years or more and less than 15, and of the domestic animals they owned, their resting places at night, and whether chickens, dogs and cats were allowed indoors or in the verandah. We requested information on chickens' current and previous nesting sites and sought direct evidence on their current nesting and resting sites.

Four teams, each one composed of one member of the research team and three skilled bug collectors, searched for triatomine bugs in all sites using timed manual collections with a dislodging spray (0.2% tetramethrin, Espacial 0.2, Buenos Aires). One person searched systematically in human sleeping quarters during 30 min (0.5 person-hour per domicile) whereas two persons searched peridomestic structures. Searches were usually conducted between 8:00 and 14:00 hours on non-rainy days, but sometimes they had to start later because householders were not available.

The weather station measured the maximum temperature during each 15-minute interval. It then reported the two-hour mean maximum temperature as the average of these eight measurements. We calculated the mean maximum temperature from 20:00 to 6:00 h as the grand mean of the mean maxima recorded in each two-hour period (data in Table S1). Our mean maximum temperatures averaged 25.4°C (SD, 3.0; range of two-hour period means, 21.9–30.0°C) and wind speed averaged 3.8 km/h (SD, 2.1; range of two-hour period means, 1.6–7.8 km/h). At 20:00 h (when bug flight and host-seeking activity usually starts or peaks), maximum temperatures averaged 31.9°C (SD, 4.9; range, 26.8–39.8°C) and wind speed averaged 5.2 km/h (SD, 3.4; range, 1.6–11.3 km/h). Therefore, weather conditions during the period from 20:00 to 6:00 h preceding every bug collection day were suitable for blood-feeding.

All triatomine bugs collected were stored in labeled plastic bags and kept in a cooler at 10–12°C between capture and arrival at the field laboratory to stop excretion, and then were identified to species and counted by stage [39]. Timing of bug catches and time of processing of the bugs from each study house were recorded. Late stages (fourth- and fifth-instars and adults) were individually examined for the presence of colorless (transparent or clear) urine within 8 hours of capture using the method developed by Catalá [34]. The abdomen of each individual bug was compressed with tweezers on a clean slide until obtaining a drop of feces whose color was recorded. We excluded first- to third-instar nymphs from the analyses because there was no information on how long transparent urine was detectable after blood-feeding.

To estimate daily blood-feeding rates, the house-specific proportion of *T. infestans* that fed during the preceding night was estimated as a weighted average of the observed proportion of fourth- and fifth-instar nymphs with colorless urine multiplied by a temperature-dependent correction factor *c* = 0.0533 * *t*–0.585 and the (uncorrected) proportion of adult bugs with colorless urine [34]. Temperatures (*t*°C) were calculated from the mean of maxima recorded every two hours between 20:00 h of the day preceding capture to the catch time. The temperature-dependent factor *c* allowed for extended detection of transparent urine in nymphs over two nights or so within the temperature range 18–28°C. In adult bugs, the transparent urine did not last from one day to the next. Specifically, if we collected and examined *x* fourth- or fifth-instar nymphs, of which a proportion *f* had transparent urine, and *y* adult bugs, of which a proportion *g* had transparent urine, then the temperature-adjusted overall proportion of late-stage bugs with transparent urine in this site was estimated as *h* = (x*c*f+y*g)/(*x*+*y*). The feeding interval (in days) was calculated as the reciprocal 1/*h*, assuming independence in feeding among bugs, a constant probability of feeding on any given day, and recently-fed bugs equally likely to be caught as not recently-fed bugs. Then a weighted average of *h* was estimated over all sites which had at least six insects examined for urine transparency. We estimated median feeding intervals because a few sites had no bugs with transparent urine, and therefore 1/*h* was indefinite; in these cases feeding intervals were arbitrarily taken to be very long (30 days) and medians calculated. All bugs were kept frozen at −20°C upon arrival to the laboratory in Buenos Aires.

### Identification of blood meals

The individual blood meals of all late-stage bugs collected at each house were prepared for testing by cutting the thorax transversally at the level of the third pair of legs and then extracting the midgut with the blood meal into a previously labeled, weighed vial [40]. Our previous studies based on some 2,000 identified blood meals of domestic *T. infestans* found minor differences among nymphal instars and adult stages [26]. Bug stages were verified on dissection and errors detected in field data sheets corrected. A direct ELISA assay was used to test bloodmeal contents against human, dog, cat, chicken, pig, goat and murid rodent (rat or mouse) antisera as described before [40]. Each microtiter plate contained test and control samples of the target host species and four negative controls (i.e., bloodmeal contents or serum of heterologous host species in PBS). Samples were tested in duplicates, and the outcome was considered valid if the mean of both duplicates of each sample did not differ by more than 15%. For each host-ELISA system, dilutions of heterologous sera were added to the antibody-enzyme conjugate solutions to decrease cross-reactivity as described in [40]. To describe how bugs fed on the available hosts, we report the proportion of reactive bugs (i.e., those positive against any of the tested antisera) that contained each type of host blood.

Sensitivity and specificity of the direct ELISA for the target host species was evaluated by testing three batches of 44 blood meals each from bugs fed to repletion separately on a dog, cat and chicken, kept at room temperature, killed 7–30 days after feeding and held frozen at −20°C; sensitivity was 100% and specificity 97–100% [40]. Additionally, we tested 5–10 sera from each of the seven target host species using the same procedures; sensitivity and specificity were 100% for all hosts. Maximum homologous serum titers at which a positive result was obtained (i.e., sensitivity limits) were higher than 200,000 for all the antisera.

### Data management and analysis

The daily rate of feeding on humans only per late-stage domestic bug was calculated as the proportion of human-fed bugs with unmixed blood meals among all domestic bugs examined for transparent urine, any bloodmeal content and bloodmeal source. Bugs were coded as 1 if they had an unmixed human blood meal and a recent blood-feed, and 0 otherwise (i.e., any bug that did not have transparent urine or a human blood meal or which was empty on dissection automatically scored as 0). Three bugs with transparent urine and mixed blood meals including humans were excluded because it could not be established whether the most recent blood meal was on humans or not; 319 bugs (from 45 houses) had valid data for human-feeding rates. The daily human-feeding rate per domestic late-stage bug divided by the total number of resident humans at each household yielded the average daily human-feeding rate per person per late-stage domestic bug.

To estimate the average number of human-bug feeding contacts per person-day in each study household, we multiplied the average human-feeding rate (per person-day per late-stage domestic bug) with the abundance of late-stage bugs (per unit of catch effort) divided by bug capture efficiency. We assumed tentatively that bug capture efficiency is the same, whether or not a bug fed on people, and that ½ person-hour would catch 10% of late stages, because one person-hour of catch effort by qualified bug collectors using a dislodging spray captured 10% of the total domestic population of *T. infestans* (capture efficiency) as determined by subsequent partial house demolition [41, based on unpublished results in the same province under similar weather conditions].

The binomial response variables analyzed were: i) daily blood-feeding rate (measuring the occurrence of transparent urine relative to the number of bugs examined for urine color, adjusted for temperature-dependent detection of transparent urine in late-stage nymphs); ii) human blood index (i.e., the proportion of reactive bugs with human blood meals detected, regardless of other blood sources), and iii) daily human-feeding rate per person per bug (as defined above). All proportions reported have attached standard errors clustered by household as estimated by Stata 12 [42].

We used a multimodel approach with model averaging run in R (version 2.15.2) [43] following the strategy outlined by Burnham & Anderson [44] and methods detailed by Grueber et al. [45] with packages lme4 (version 0.999999-2), MuMIn (version 1.9.5), arm (version 1.6–06.01), ResourceSelection (0.2–2), and ROCR (version 1.0–5). For this purpose we fitted GLMMs to a global model that included all the explanatory variables. The random-intercept regression models clustered by bug collection site addressed the fact that insects from a given site shared the same environment and other undetermined characteristics that may have created dependencies among responses within the same cluster of observations. All predictors were established a priori based on existing empirical evidence reviewed elsewhere [2], [40]. The global model fitted to the response variables (p*_ij_*) was:

logit (p*_ij_*) = ln (p*_ij_*/(1-p*_ij_*)) = α+β_1_×chickbi*_j_*+β_2_×dogbi*_j_*+β_3_×humtot*_j_*+β_4_×stage3*_ij_*+β_5_×maxtemp*_j_*+β_6_×bugabund*_j_*+a*_j_*,

where α is a constant; β_1_-β_6_ are regression coefficients; subscripts are for insect *i* on house *j*; chickbi is the chicken blood index for house *j* (a proportion); dogbi, the dog blood index for house *j* (a proportion); humtot, the total number of human residents per house; stage3, bug stage (with three levels: fourth- or fifth-instar nymphs; adult males, and adult females); maxtemp, mean maximum temperatures during the night preceding bug catch; bugabund, domestic bug abundance per unit of catch effort (including any first-third instar nymphs caught); a*_j_*, the random intercept term, assumed to be independent across houses and normally distributed with mean 0 and variance σ_a_^2^ [46]. Predictors were not transformed for analysis.

We investigated potential multicollinearity problems of the standardized variables (except stage) via variable inflation factors (VIF), R^2^ and the condition number. For every predictor, VIFs were <1.25, R^2^<0.2, and the matrix had a condition number of 3.2, indicating no significant multicollinearity. For additional analyses, we added the square of bug abundance to the global model to test for non-linear effects; marginally significant effects were detected only for daily feeding rates (results not shown). We also replaced the chickbi and dogbi with the reported or observed occurrence of chickens indoors and the number of dogs sharing human sleeping quarters (“roommate” dog) per household, respectively, while keeping all other predictors the same.

For model selection we used Akaike's Information Criterion corrected for small samples (AIC_c_). The subset of models that were within 4 AIC_c_ from the best-fitting model were considered the top models. The relative importance (RI) of each explanatory variable in the top model set was computed using the Akaike weight for each model in which the variable was present. The overall quality of the fitted logistic regression models was assessed by means of the Hosmer and Lemeshow test using the averaged coefficients, and by the area under the receiver operating characteristic curve (ROC). The latter can be interpreted as the fraction of all observations that are correctly classified by the model.

We compared the outcome from the multimodel framework with the more traditional approach based on null-hypothesis testing via random-intercept logistic regression models clustered by bug collection site as implemented in the command xtlogit in Stata 12 [46]. Regardless of the response variable, the statistically significant predictors in the random-intercept models consistently had large RI (chicken and dog blood index) in the multimodel approach. To simplify the exposition, we report only the multimodel analysis in the main text and show estimates based on null-hypothesis testing in Table S3.

## Results

### Population abundance

Of 543 bugs from all stages collected at 94 houses with infested human sleeping quarters, 17.7% were first- to third-instar nymphs; 12.7% fourth instars; 26.3% fifth instars; 21.2% females, and 22.1% males. Domestic bug abundance (median, 5 bugs per 0.5 person-hour) was highly variable (first-third quartiles, 2-9 bugs; maximum, 41 bugs) at the 49 study houses and highly overdispersed (variance-to-mean ratio, 91.3/8.1 = 11.3). A median study household had 5 people (first-third quartiles, 4–8), 2 dogs (2–3), 0.5 cat (0–1), 15 chickens (7–22), 11 goats (0–29), 2 pigs (1–6) and 2 horses (0–4); very young chickens were recorded in 71% of houses. The proportion of the 49 study households that reported allowing animals to sleep indoors was 55% (dogs), 37% (cats) and 35% (chickens). Chickens were allowed to roam freely, nesting inside domiciles, kitchens, storerooms, chicken coops, or almost anywhere around human sleeping quarters.

Bug abundance correlated positively and significantly with numbers of dogs (r = 0.411, P<0.004) and cats (r = 0.291, P<0.05), marginally with the total number of chickens (r = 0.280, P<0.06), and non-significantly with human residents (r = 0.076, P>0.6) or the indoor presence of chickens (r = 0.136, P>0.3).

### Blood-feeding rates

**Figure 1:**
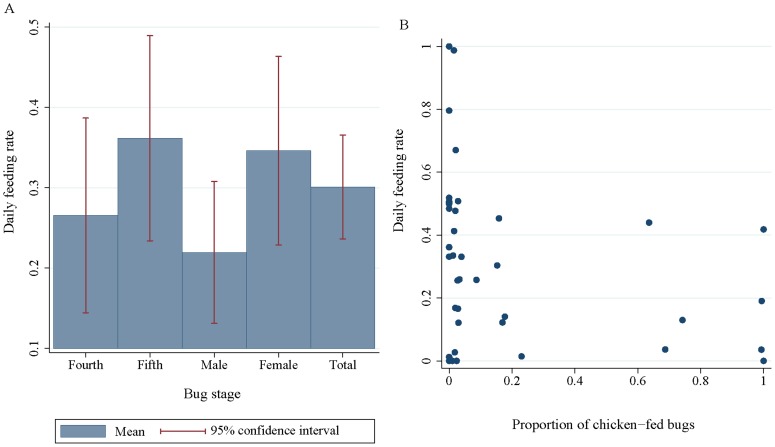
Temperature-adjusted proportion of domestic *T. infestans* that blood-fed the night before catch according to bug stage (A) and the chicken blood index (B). Figueroa, October 2003 (spring). In B, each data point corresponds to a different house.

Of 326 late-stage insects (from 49 houses) that were processed, 319 were examined for transparent urine. The percentage of late-stage domestic *T. infestans* with transparent urine averaged 33.9% (108 of 319, 95% Confidence Interval [CI], 27.1–40.6%). Temperature-adjusted feeding rates were nearly 4% lower than unadjusted values (30.0%, CI, 23.6–36.6%). Daily blood-feeding rates increased from 26.6% in fourth-instar nymphs to 36.2% and 34.6% in fifth instars and females, respectively, and declined to 21.7% in males (Figure 1A). Median blood-feeding intervals were 4.1 days, with large variations among households (first and third quartiles, 2.4-7.0 days). A simple sensitivity analysis showed that a 10% perturbation of the parameters in the formula for the correction factor *c* translated into close values for the daily feeding intervals at 3.4 and 5.0 days (Text S1).

Daily blood-feeding rates decreased as the chicken blood index increased (OR = 0.48, CI, 0.26-0.89), with marginal effects of male stage (OR = 0.58, CI, 0.30–1.11)(Table 1, Figure 1B). There was large model uncertainty (Table S4) and no empirical support for effects due to mean maximum temperatures during the night preceding bug capture (Figure S1), bug abundance, the dog blood index and number of human residents (Table 1 and S1). The averaged model fitted the data well (Hosmer and Lemeshow χ^2^ test = 5.68, 8 df, P>0.6), and the area under the ROC was 0.615, thus indicating a poor classification. None of the predictors had significant regression coefficients when the chicken and dog blood indices were replaced with the reported or observed presence of chickens indoors and the household number of roommate dogs, respectively (not shown).

**Table 1:** Model-averaged coefficients of factors associated with the daily blood-feeding rate, human blood index, and daily human-feeding rate of domestic *T. infestans* in Figueroa, spring 2003.

	Daily blood-feeding rate^a^				Human blood index^b^				Human-feeding rate^c^			
Variable^a^	Coefficient	S.E.	P	RI	Coefficient	S.E.	P	RI	Coefficient	S.E.	P	RI
Intercept	−0.8235	0.1750	***		1.1690	0.2386	***		−1.6694	0.2623	***	
Chicken blood index	−0.7294	0.3110	*	0.95	−4.5767	0.5907	***	1.00	−3.0073	0.7973	***	1.00
Dog blood index	−0.3416	0.2863	ns	0.44	−2.3817	0.4216	***	1.00	−0.9692	0.3713	**	1.00
Stage: males	−0.5625	0.3336	ms	0.45	0.2003	0.5135	ns	0.06	−0.5178	0.3761	ns	0.31
Stage: females	0.0753	0.3157	ns	0.45	0.4139	0.5321	ns	0.06	0.1117	0.3456	ns	0.31
No. of humans	0.3704	0.2821	ns	0.39	−0.0427	0.4664	ns	0.25	0.0206	0.2884	ns	0.19
Bug abundance	0.1743	0.2857	ns	0.26	−0.6340	0.4815	ns	0.42	0.1910	0.2681	ns	0.28
Maximum temperature	−0.1022	0.2884	ns	0.24	−0.8591	0.5075	ns	0.60	−0.0727	0.2892	ns	0.20

*** P<0.001; * P<0.05; ms, 0.05<P<0.1; ns, P>0.1.

SE, standard error; RI, relative importance.

a Total number of observations with complete data for every predictor: 317 bugs from 47 houses.

b Total number of observations with complete data for every predictor: 289 bugs from 45 houses.

c Total number of observations with complete data for every predictor: 314 bugs from 44 houses.

### Host-feeding choices

**Figure 2:**
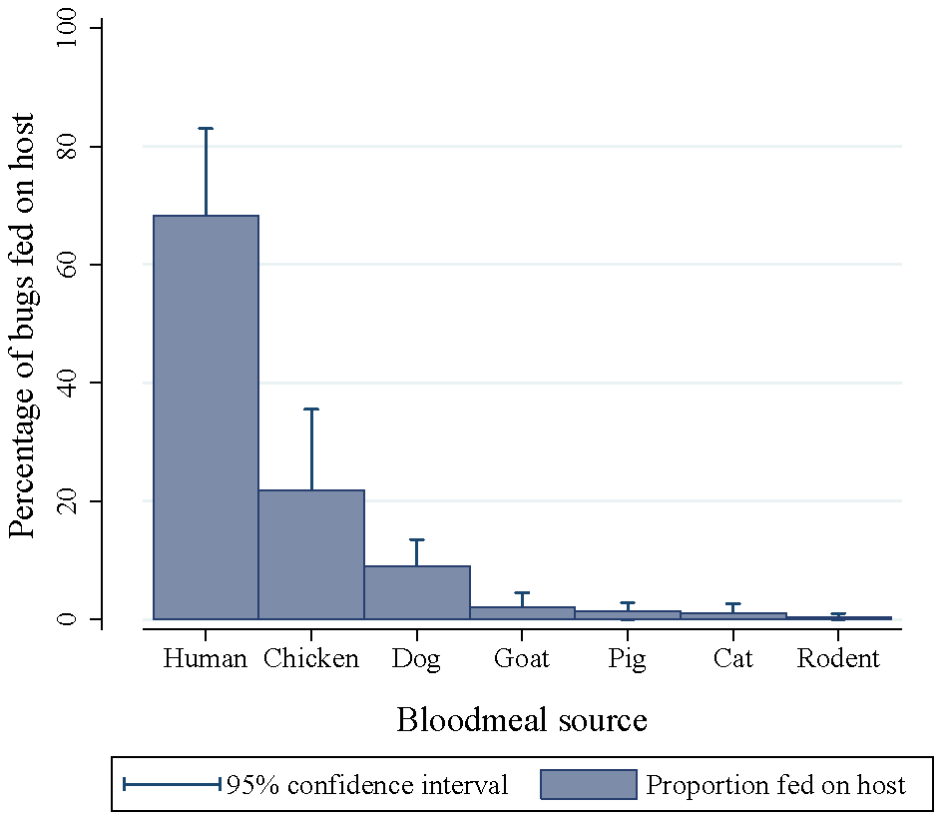
Percentage of late-stage *T. infestans* collected in human sleeping quarters that fed on each host species (regardless of feeding on other host species). Figueroa, spring 2003. 95% confidence intervals clustered by site.

All 319 *T. infestans* examined for transparent urine were dissected and some blood meal residue extracted from 305 bugs (from 47 houses). At least one host bloodmeal source was identified in 289 (94.8%) of the bugs (from 45 houses); these 289 bugs were termed “reactive” (bloodmeal identification data in Table S2). The main bloodmeal sources were humans (68.2%, 95% CI, 53.4–83.0%) followed by chickens (21.8%, 8.2–35.5%) and dogs (9.0%, 4.5–13.5%) (Figure 2). Blood meals on goats (2.1%, 0.4–4.5%), pigs (1.4%, 0–2.8%), cats (1.0%, 0.5–2.6%) and rodents (0.3%, 0–0.1%) were rare. Most reactive bugs had unmixed meals (95.2%), a few had fed on two host species (4.5%) and hardly any on three (0.3%). The proportion of bugs collected from human sleeping quarters with unmixed blood meals from humans was very high (65.4%, CI, 50.4–80.4%) compared with the series of 16 similar studies compiled in [26]. The mean proportion of chicken-fed domestic bugs clustered by site was five times greater in houses where the indoor presence of chickens was reported or observed (43.3%, CI, 18.7–68.0%) than in those where it was not (8.0%, CI, 0–18.9%). Among all the reactive bugs collected, the selection of human versus chicken (or dog) bloodmeal sources was highly significantly associated (Fisher's exact tests, P<0.0001).

### Human-feeding rates

**Figure 3:**
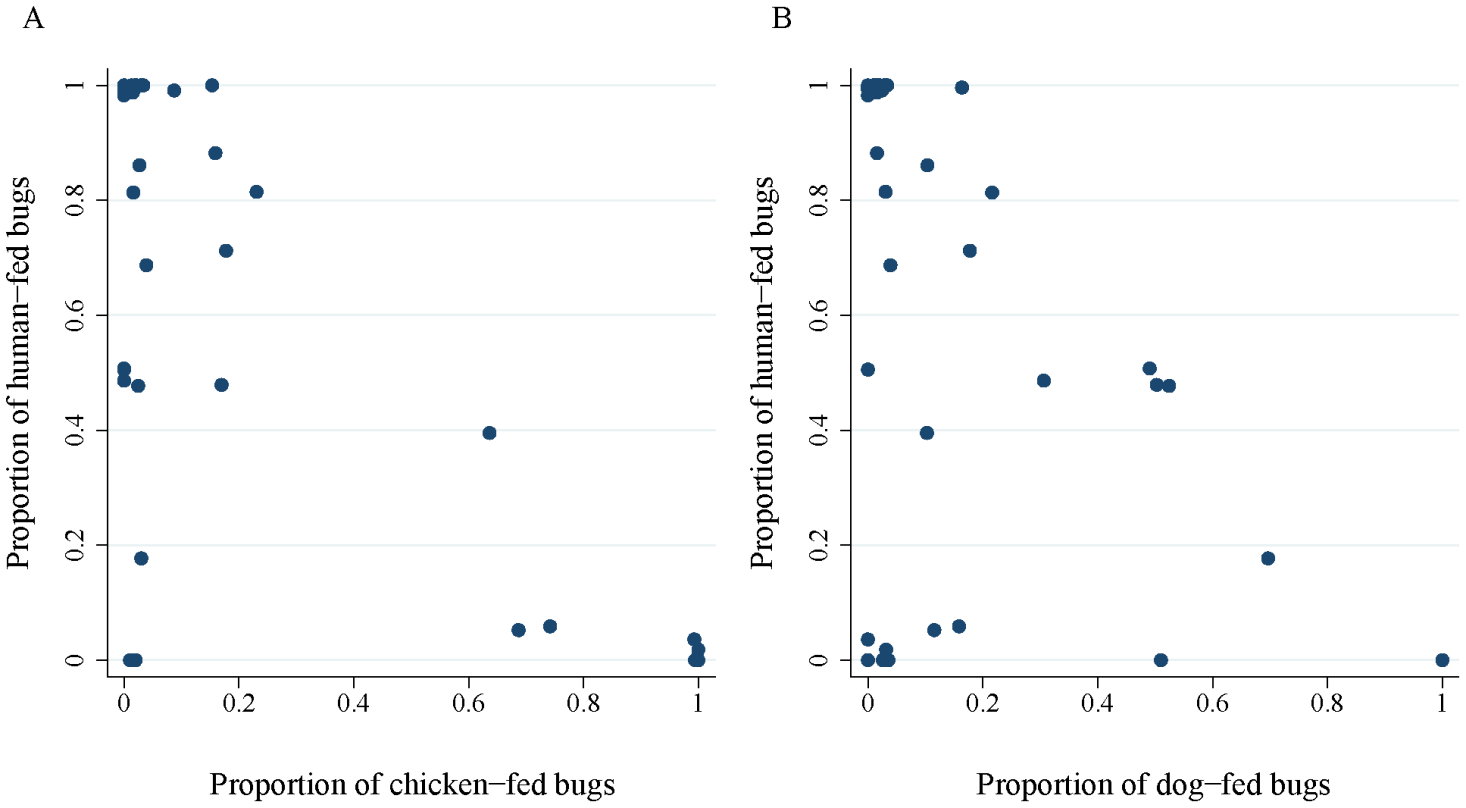
Proportion of late-stage domestic *T. infestans* that fed on humans (the human blood index) according to the proportion of bugs that fed on chickens (A) and dogs (B). Each data point corresponds to a different house. The human and chicken blood indices include bugs that fed on humans or chickens, respectively, regardless of feeding on other species. Figueroa, October 2003 (spring).

The proportion of human-fed bugs decreased strongly with an increasing chicken blood index (OR = 0.010, CI, 0.003-0.033) and with an increasing dog blood index (OR = 0.092, CI, 0.040–0. 211) (Table 1, Figure 3). There was no evidence that the number of resident humans, bug stage, mean maximum temperatures during the night preceding bug capture, and bug abundance per site exerted any effects on the human blood index (Table 1 and S1). The averaged model fitted the data very closely (Hosmer and Lemeshow χ^2^ test = 9.8, 8 df, P>0.28), and the area under the ROC was 0.941 −an outstanding classification. Replacing the chicken blood index with the presence of chickens indoors and the dog blood index with roommate dog numbers yielded significant regression coefficients for the indoor presence of chickens (OR = 0.075, CI, 0.008–0.720) and marginally positive effects of the number of human residents (OR = 12.72, CI, 0.86–188.99). Hence, the indoor presence of chickens and dogs translated into a lower human blood index.

The mean human-feeding rate per 100 bug-days was estimated as 23.1 and the overall mean number of human-bug feeding contacts per person-day as 7.2 (Table 2). When chickens were available indoors, percent human-feeding rates were substantially lower both in small and large (14.7–15.8) households than when chickens were not reported or observed indoors (27.1–30.7) (Kruskal-Wallis, df = 1, P<0.007), but there was large variation within any group of households. The indoor presence of chickens was also associated negatively with the frequency of human-feeding contacts (Kruskal-Wallis, df = 1, P<0.001). Despite having lower bug abundance, small households without chickens indoors (42% of all infested houses) would experience two to five times more human-bug feeding contacts per person-day (12.7) than other households (2.7–5.2) in spring. One small household without chickens indoors had an extreme value of 46.7 human-feeding contacts per person-day. Excluding this extreme value did not alter the qualitative relations between human-feeding rates or frequency of feeding contacts with household size and indoor presence of chickens.

**Table 2:** Human-feeding rate per 100 bug-days and number of human-bug feeding contacts per person-day of late-stage domestic *T. infestans* according to presence of chickens indoors and numbers of resident people (Figueroa, spring 2003).

No. of people	Presence of chickens indoors	No. of houses^a^	Mean bug abundance per person-hour (SE)	Human-feeding rate per 100 bug-days (CI)	No. of human-bug feeding contacts per person-day (CI)
1–5	Yes	7	8.7 (2.6)	14.7 (0.2–29.2)	4.0 (0.2–7.9)
1–5	No	20	7.3 (2.1)	30.7 (21.1–40.2)	12.7 (0–30.3)
6–17	Yes	10	10.6 (4.1)	15.8 (0.1–43.3)	2.7 (0–6.6)
6–17	No	11	7.5 (2.6)	27.1 (10.9–43.3)	5.2 (2.6–7.7)
Total		48	8.1 (1.4)	23.1 (15.7–30.5)	7.2 (0.3–14.2)

a Excludes one house with no human occupants.

CI, 95% confidence interval.

**Figure 4:**
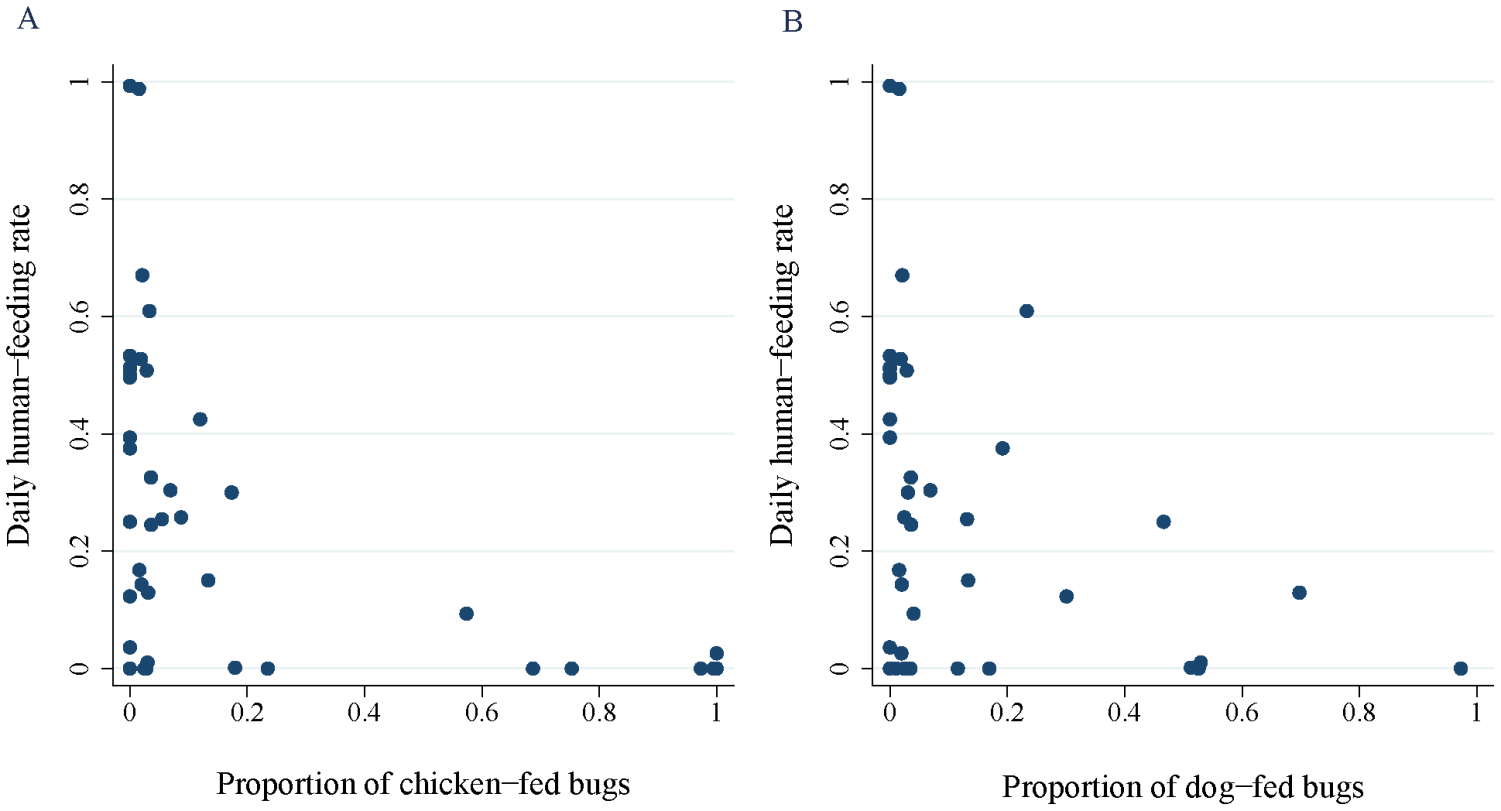
Daily human-feeding rate of late-stage domestic *T. infestans* according to the proportion of chicken-fed bugs (A) and dog-fed bugs (B). The daily human-feeding rate is the proportion of bugs that fed on humans only on the previous night. Figueroa, October 2003 (spring). Each data point corresponds to a different house.

The daily human-feeding rate also decreased dramatically with increases in the chicken blood index (OR = 0.049, CI, 0.010–0.236) and dog blood index (OR = 0.379, CI, 0.183–0.786) (Figure 4, Table 1). Other predictors exerted no significant effects (Table 1 and S1). The averaged model fitted the data very closely (Hosmer and Lemeshow χ^2^ test = 6.8, 8 df, P>0.5), and the area under the ROC was 0.740 –an acceptable classification level. The indoor presence of chickens was associated with a lower daily human-feeding rate (OR = 0.414, CI, 0.191-0.900).

## Discussion

Very high human blood indices and human-feeding rates of domestic *T. infestans* decreased significantly with the increasing frequency of blood meals on chickens and dogs or the occurrence of chickens indoors in spring, in support of hypothesis 2 (i.e., the human blood index and human-feeding rate should decrease with more chicken or dog blood meals and the occurrence of indoor-nesting chickens and dogs). As predicted by a mathematical model of transmission, the data most strongly supported the hypothesis 4 that the rate of human-bug feeding contacts per person-day decreased substantially with the indoor presence of chickens and dogs [31].

These results –derived from combining information on daily feeding rates and host blood indices in domestic areas with household-level demographic data– support and extend previous findings and predictions: high human blood indices in domestic *T. infestans* were inversely related to chicken and dog blood indices in surveys conducted elsewhere in Santiago del Estero during spring and summer [2]. The large fraction of bugs with unmixed blood meals implies that the bugs fed repeatedly on the same domestic host species (host fidelity), which is consistent with previous observations and the usual finding of bugs in the proximities of host resting sites. Such increased human exposure is explained by nightly resting patterns in winter and spring, when most local villagers still slept indoors. In this region, chickens frequently are allowed to nest indoors or are deliberately introduced there for their protection; they have constant nesting locations during a few weeks favoring bug feeding on them, and peak nesting frequency in spring and early summer [47].

During the hot summer, however, people increasingly moved their beds to sleep in the verandah or patio (which reduced their exposure) and domestic bugs fed more often on dogs and chickens, which reduced the human blood index. In the current study we confirm that the same qualitative relationships hold in other rural communities when domestic infestations were lighter and dogs were less important bloodmeal sources than in earlier studies, and extend those results to human-feeding rates.

Domestic bugs fed mostly on humans and chickens and had fewer dog blood meals or mixed blood meals than in other studies conducted in this region during late winter-early spring [26]. Differences in host exposure patterns and domestic animal husbandry practices among settings may account for such variations, given that bloodmeal identification methods (ELISA versus agar double-diffusion tests) had similar performance in a large-scale comparative trial of mosquito blood meals [48]. Compared with 16 published studies of domestic *T. infestans*, the observed human blood index (68%) ranks tenth and equals the mean human blood index found in the same study region in Santiago del Estero over 1988-1992 [26]. The fraction of unmixed blood meals in the current study (95%) is substantially higher than the average across the 16 published studies (70%), and as a consequence, the fraction of bugs with unmixed human blood meals is comparatively very high (65.4%) [26].

Our study provides an improved way of estimating the daily host-feeding rate of triatomine bugs and a measure of its precision that are applicable to other settings and species of Triatominae. The product of the human blood index (including both unmixed and mixed blood meals) and daily blood-feeding rate is not dimensionally consistent; it is not a daily rate because the bugs may have fed on humans (or on any other host species) over the usually long period of time in which blood meals are detectable by immunologic methods (i.e., up to three or four months, depending on initial and subsequent bloodmeal sizes and temperature [49 and references therein]), and because the bugs may have fed once or several times [26]. Therefore, the human-feeding rate calculated on the basis of all human blood meals (mixed and unmixed) is biased upward relative to the true rate because it may include older human blood meals. The magnitude of the bias is expected to increase with the proportion of mixed blood meals, which in domestic *T. infestans* usually increases from a minimum in late winter-early spring (as in the current study) to late summer, and is quite high throughout the species' range [26], [50]. Conversely, the unmixed human blood index among bugs that fed on the preceding night is a more accurate estimate of the daily human-feeding rate. To reduce the potential bias and detect mixed blood meals, at least all resident host species in the study habitats need to be included in sensitive bloodmeal identification tests. These considerations also apply to the derived human-feeding rate per person.

Our estimate of the average blood-feeding interval of domestic *T. infestans* in spring (4.1 days) is higher than another spring-summer average (3 days) [35], and considerably lower than another estimate for domestic adult bugs (5–8 days) in two heavily infested houses [33]. This high feeding frequency is consistent with experimental work showing that when a live rabbit is continually present *T. infestans* and *R. prolixus* females will feed about every three days [51]. Moreover, there were large variations in blood-feeding rates among houses. The top models for blood-feeding rates had considerable model uncertainty (i.e., low Akaike weights) and less support than the models for the human blood index and human-feeding rate. According to the averaged model, the more the bugs fed on chickens the less frequently they fed. This result was surprising and might be related to an eventually larger bloodmeal size of bugs feeding on chickens; if bugs achieved a higher engorgement status when feeding on chickens, then they would not need to feed as often as bugs that ingested smaller blood meals. In the absence of direct measures of bloodmeal size, this explanation is tentative and the subject needs to be more widely investigated. Male bugs tended to feed less often than females [52] or late nymphal instars which require more energy for laying eggs and molting. Unlike longer experimental studies [34], [38], no temperature or host abundance effects on daily feeding rates were detected despite considerable variations in temperature among catch days (range, 13°C) and in domestic host abundance among households (range, 2–17), thus rejecting hypothesis 1. Lack of effects of bug abundance on blood-feeding rates, human blood indices and human-feeding rates (hypothesis 3) may be explained by the rather limited range of variation in domestic bug abundance at the beginning of the reproductive season a few years after vector control actions, and the imprecision of bug abundance estimates by timed manual collections. The evidence collectively indicates that the high blood-feeding rate of domestic *T. infestans* is little affected by temperature, stage, numbers of domestic hosts and domestic bug abundance in this specific context in the dry Argentine Chaco spring.

The estimated number of human-bug feeding contacts per person-day averaged 7.2 (median, 3.6) over households and was quite high. If the bugs bite only at night, this means nearly one human-bug feeding contact per person every one to two hours during the night, but more extreme values were recorded. These estimates do not include first- to third-instar nymphs, which comprise an increasingly larger fraction of the bug population from spring to summer. First- to third-instar nymphs are easily captured in human beds, and most are fed on humans [26], [28], [41]. These small instars may thus increase substantially the total human-bug contact rate, ensuing blood loss, host irritation, and inoculation of bug saliva with potentially allergic effects [53]. Uncertainty about the capture efficiency of bugs per unit of effort limits the absolute value of estimates for each of these quantities, but still allows a direct comparison among households. For example, the proportion of existing late-stage domestic *R. prolixus* collected manually without a dislodgant spray varied from 4 to 41% depending on bug stage and type of walls and roof, and was invariant with absolute bug abundance [54]. The estimated daily number of human-bug feeding contacts also puts a cap on the frequency of potentially infective contacts experienced by an average person because late–stage bugs also concentrate most infections with *T. cruzi* [25], [55].

Our study has both limitations and strengths. The estimated blood-feeding intervals may be taken as good approximations to the actual values. In addition to the pioneering studies of Catalá [34] using live pigeons, our subsequent experiments using heparinized cow blood and an artificial feeding apparatus showed that transparent urine was detected regardless of bloodmeal size in >95%, 20% and none of a total of 729 fourth- and fifth-instars nymphs of *T. infestans* held at 26–28°C and examined at 12, 36 and 60 h post-feeding, respectively (P. L. Marcet et al., unpublished results). These results are in close agreement with the predicted values at 25°C [34] −the average overnight temperature during the current field study− despite major differences in host species, host blood composition (i.e., bird versus mammal) and experimental setup. Our experiments also showed that transparent urine was detectable at 12 h post-feeding in 90% of adult females that had achieved full blood meals, and then persisted only in 4% of them at 36 h post-feeding (total examined, 285 females); partial blood meals slightly reduced the rate of occurrence of transparent urine after feeding. The achieved bloodmeal sizes of adult bugs under field conditions are unknown and may be used to improve estimates of blood-feeding. This method would not be able to reveal the occurrence of multiple blood meals during the same night. *T. cruzi* infection does not modify the rate of urine excretion in triatomine bugs, and affects minimally their vital rates only under severe starvation conditions [56], [57], which was not the case for the study bug populations. The experimental effects of *T. cruzi* infection on blood-feeding rates and blood intake of *T. infestans* were minor, temperature-dependent and confined to fifth instars [56], [58]. Although the correction factor *c* was calibrated in the range between 18 and 28°C whereas overnight average maximum temperatures sometimes exceeded 30°C, the linear relationship between *c* and temperature has strong underlying physiological foundations: The rate of urine excretion (diuresis) in *R. prolixus* was constant and linearly related to temperature within the range 17–30°C, and responded almost immediately to temperature changes [59].

Although molecular methods represent an improvement over ELISA for bloodmeal identification at host genus or species levels, different PCR protocols frequently fail to detect host bloodmeal sources in a sizable (15–60%) proportion of tested bugs [e.g.,50,60,61] and older blood meals. This is likely related to degradation of host DNA during the blood meal's long periods of storage in the anterior midgut. Unlike serologic methods for bloodmeal identification [49], genus- or species-specific PCRs used to detect the persistence of host DNA in *T. infestans* experimentally fed on major domestic host species identified host sources consistently only up to two weeks post-feeding, and to a much lesser extent up to four weeks post-feeding [62]. For domestic bug populations exposed to a limited, well-known range of domestic host species, the ELISA test is a cost-effective option showing high sensitivity and specificity combined with high-throughput processing. It revealed bloodmeal residues that were visually undetectable and a sizable fraction of mixed blood meals on dogs and chickens or cats housed overnight in pairs in experimental huts [40]. Here the ELISA test kept the proportion of non-reactive bugs below 5%, yet it may have missed marginal blood meals on other infrequent hosts (e.g., horses). Future studies may benefit from collecting larger samples of bugs per site to increase the precision of estimates.

Host occupancy changes over time and is difficult to gauge precisely [37], especially in peridomestic sites, which limits our understanding of host-feeding choices and related metrics. The question of which factors are causing host shifts or the lack of them (e.g., host availability and accessibility, bug attack rates, temperature, refuge availability and its interactions) remains unresolved. Major strengths of this study are the detailed information on various bug attributes in the identified sleeping quarters of a sizable number of houses from a well-defined rural area, and appropriate GLMM-based modeling of clustered data in a multimodel inferential framework.

### Implications for vector control and disease transmission

We have not assessed the links between host-feeding rate or host choice and household infection with *T. cruzi*, a central goal that requires a much greater research effort. However, some evidence allows us to make inferences about parasite transmission. Our study shows a very intense human-bug contact rate in spring that is consistent with the peak of monthly acute cases of Chagas disease recorded historically in October and November in northern Argentina [63], [64]. Other field and experimental data also showed that the abundance of domestic *T. infestans*, its prevalence of infection with *T. cruzi*, and the mean intensity of parasites in bugs' rectal contents increased very fast from a minimum at the end of winter to high or submaximal values by the end of spring [25], [64], [65]. Average temperatures during our study are considered optimal for *T. infestans*. Because domestic bugs feed disproportionately more on humans at the beginning of the reproductive season in late winter and spring than during the hot summer season when bug density peaks [31], spring is a high-risk period for increased domestic transmission of *T. cruzi*.

Although a snapshot of the transmission system indicates that indoor chickens and dogs in the spring lower (at least temporarily) the probability of vectors feeding on transmission-competent hosts and human-bug feeding contacts (supporting hypotheses 2 and 4), it fails to capture the entire range of dynamic effects on parasite transmission rates over time. The indoor presence of dogs in spring may increase human risks of infection because the infectivity of *T. cruzi*-infected dogs is 18.7 times greater than that of infected humans [21]. The increased spring bug population fed on indoor-resting chickens, humans and dogs [28] would increase the human-bug contact rate during the subsequent summer when the nesting activity of chickens declines and chickens move away from human sleeping quarters [25], [31] while most people move outside to sleep in their verandahs. Based on this chain of effects, each individually well documented, we hypothesize (as confirmed by 1992 summer data [25], [29] from Amamá, Santiago del Estero, Argentina, and in accordance with our mathematical model of transmission [31]) that the net effects of chickens on parasite transmission in the presence of transmission-competent hosts may be more adequately described by zoopotentiation [13] than by zooprophylaxis. This hypothesis cannot be regarded as tested in Figueroa until quantitative data from Figueroa on the prevalence and transmission of infection in bugs, humans, and other hosts during the summer months, or over an annual cycle, are analyzed.

The large variability among households in blood-feeding rates and human blood indices implies large uncertainty in the threshold human-feeding rate of domestic *T. infestans* under which no transmission would occur. Measurement of a threshold human-feeding rate for transmission of vector-borne pathogens is fraught with several sources of inaccuracy, including measurement of vector abundance, host exposure and the probability of human infection given a feeding contact with an infected vector, for which only indirect estimates are possible [5], [24], [31], [41], [66]. Estimates of transmission thresholds using house infestation prevalence at a village-wide level [67] are even more removed from the basic quantities and processes that determine the theoretical thresholds and should be regarded with reservations until solid evidence is available.

The presence of domestic animals in human sleeping quarters and changing site occupancy by different domestic host species exert profound effects on the population dynamics of *T. infestans*, host-feeding choices and ensuing risks of parasite transmission. The evidence available thus far advises against the presence of domestic animals in human sleeping quarters [24], [25], [31]. Treating chickens and dogs with insecticides that have reduced repellency may turn them into baited lethal traps and exert substantial impacts on infestation and transmission [68]. Appropriate animal husbandry combined with long-lasting environmental modifications in housing and peridomestic structures [69] and judicious use of residual insecticide spraying are crucial for sustainable vector and disease control of Chagas disease in affected rural areas [21]. The human-feeding rates of major domestic vectors of *T. cruzi* should be estimated more widely with currently available methods. Whether zooprophylaxis or zoopotentiation applies to other domestic triatomine species in other regions and ecoepidemiological circumstances merits further research.

## Supporting Information

Figure S1 **Temperature-adjusted proportion of domestic** ***T. infestans*** **that blood-fed the night before catch (daily feeding rate) according to mean maximum temperature during that night.** Figueroa, October 2003 (spring).
(TIF)

Figure S2 **Human blood index (A) or human-feeding rate (B) according to daily feeding rates of domestic** ***T. infestans*** **for all houses.** Figueroa, October 2003 (spring).
(TIF)

Table S1 **Weather data during bug collection surveys.** Figueroa, October 2003 (spring).
(XLS)

Table S2 **Bloodmeal identification results of domestic** ***T. infestans*** **as determined by direct ELISA tests.** Figueroa, October 2003 (spring). Codes 0 and 1 are for negative and positive identification results, respectively; ND, no data.
(XLS)

Table S3 **Random-intercept multiple logistic regression obtained with Stata.**
(DOC)

Table S4 **Multimodel assessment of factors associated with daily blood-feeding rates, human blood index, and human-feeding rates of** ***T. infestans*** **.** Figueroa, spring 2003.
(DOC)

Text S1 **Sensitivity analysis of blood-feeding rates to changes in parameter estimates of the correction factor.**
(DOC)
