# Supplementary figures and images for: Domestic Animal Hosts Strongly Influence Human-Feeding Rates of the Chagas Disease Vector Triatoma infestans in Argentina
Source: PLoS Negl Trop Dis. 2014 May 22;8(5):e2894. doi: 10.1371/journal.pntd.0002894 (PMC4037315; doi:10.1371/journal.pntd.0002894)

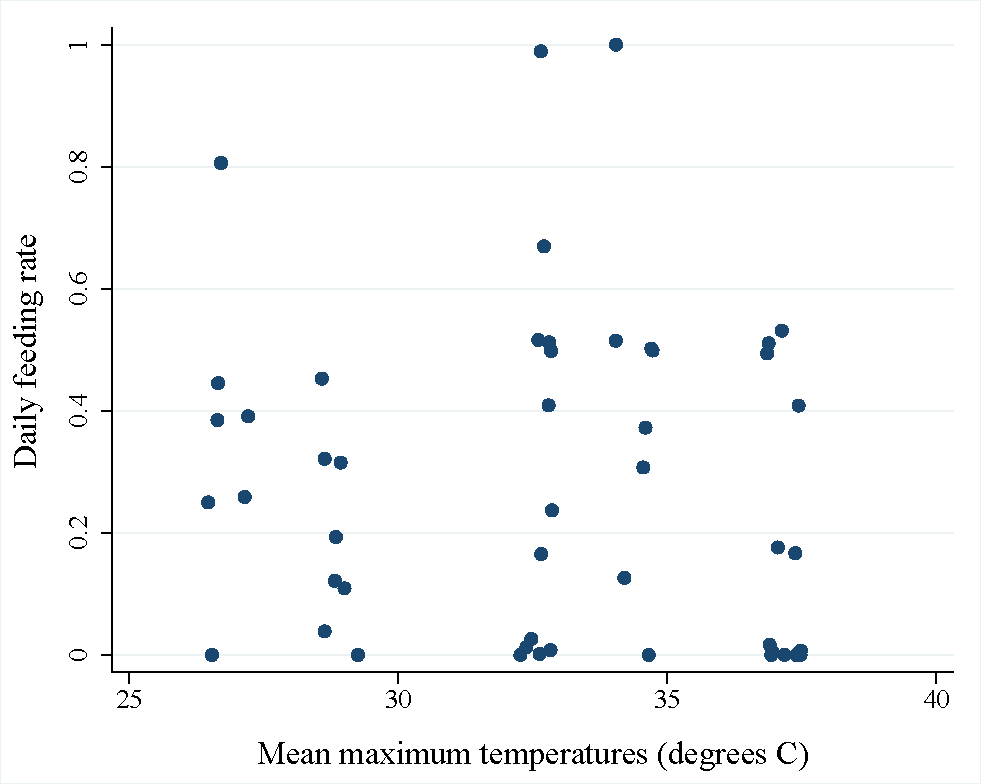

Supplement: Figure S1 — Temperature-adjusted proportion of domestic T. infestans that blood-fed the night before catch (daily feeding rate) according to mean maximum temperature during that night. Figueroa, October 2003 (spring). (TIF) [file pntd.0002894.s001.tif]

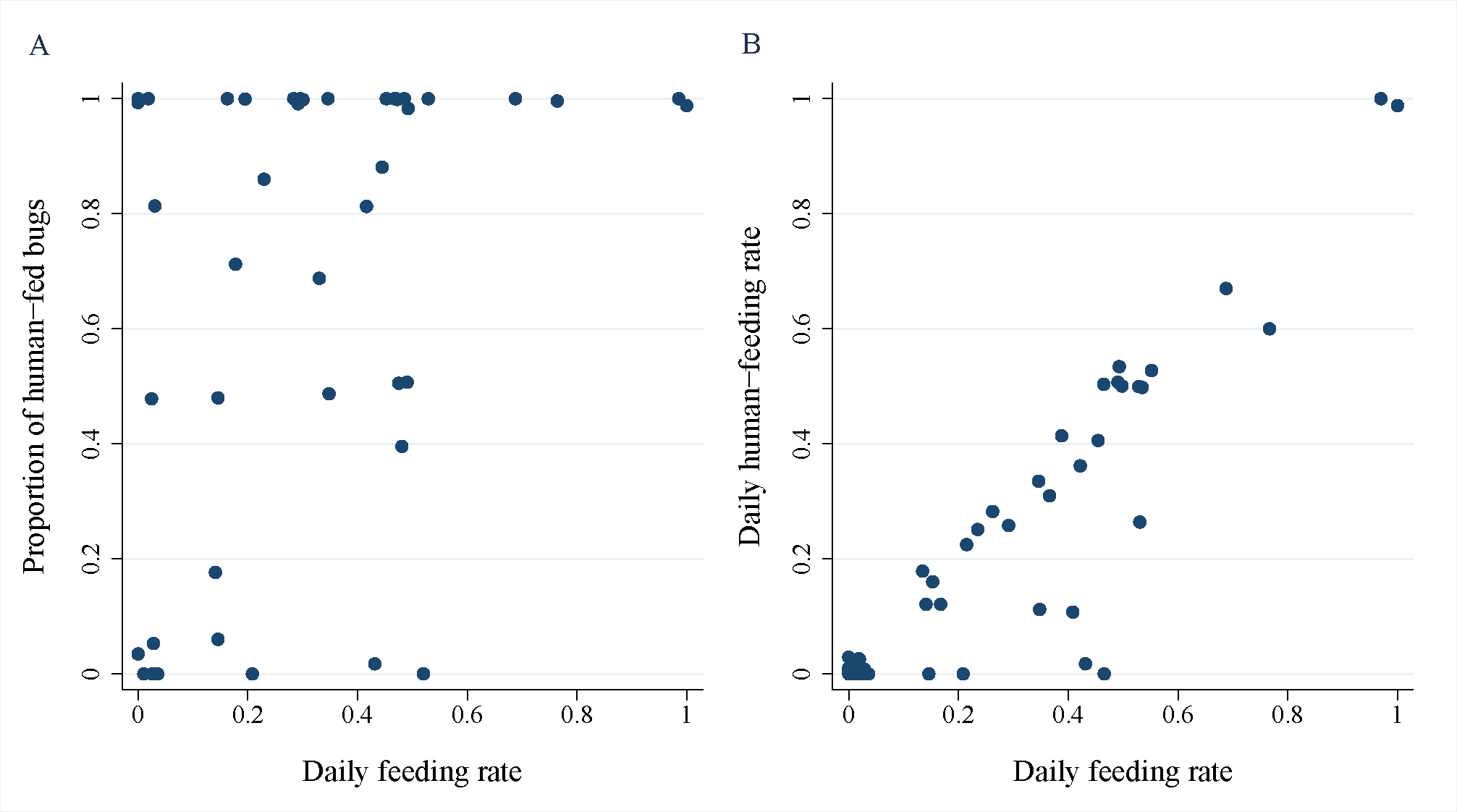

Supplement: Figure S2 — Human blood index (A) or human-feeding rate (B) according to daily feeding rates of domestic T. infestans for all houses. Figueroa, October 2003 (spring). (TIF) [file pntd.0002894.s002.tif]
