# Supplementary material for: Domestic Animal Hosts Strongly Influence Human-Feeding Rates of the Chagas Disease Vector Triatoma infestans in Argentina
Source: PLoS Negl Trop Dis. 2014 May 22;8(5):e2894. doi: 10.1371/journal.pntd.0002894 (PMC4037315; doi:10.1371/journal.pntd.0002894)
Supplement: Table S3 — Random-intercept multiple logistic regression obtained with Stata. (DOC) [file pntd.0002894.s005.doc]

**Table S3.** Random-intercept multiple logistic regression obtained with Stata, Figueroa, spring 2003.

|  | Daily feeding rate | | | Human blood index | | | Human-feeding rate | | |
| --- | --- | --- | --- | --- | --- | --- | --- | --- | --- |
|  | Coefficient | S.E. | P | Coefficient | S.E. | P | Coefficient | S.E. | P |
| Intercept | -0.1275 | 1.6166 | 0.94 | 8.0912 | 3.0662 | 0.01 | 0.0583 | 1.6487 | 0.97 |
| Chicken blood index | -1.1210 | 0.4715 | 0.02 | -6.9388 | 0.9357 | <0.001 | -4.5105 | 1.1924 | <0.001 |
| Dog blood index | -0.9826 | 0.9074 | 0.28 | -8.3197 | 1.4866 | <0.001 | -2.8574 | 1.1445 | 0.01 |
| Stage: males | -0.5709 | 0.3500 | 0.10 | -0.0799 | 0.5445 | 0.88 | -0.4854 | 0.3987 | 0.22 |
| Stage: females | 0.0695 | 0.3382 | 0.84 | 0.1092 | 0.5661 | 0.85 | 0.1639 | 0.3741 | 0.66 |
| No. of humans | 0.0524 | 0.0507 | 0.30 | -0.0153 | 0.0783 | 0.85 | 0.0048 | 0.0512 | 0.93 |
| Bug abundance | 0.0040 | 0.0120 | 0.74 | -0.0248 | 0.0199 | 0.21 | 0.0072 | 0.0117 | 0.54 |
| Maximum temperature | -0.0210 | 0.0439 | 0.63 | -0.1304 | 0.0806 | 0.11 | -0.0182 | 0.0453 | 0.69 |
