# Supplementary material for: Domestic Animal Hosts Strongly Influence Human-Feeding Rates of the Chagas Disease Vector Triatoma infestans in Argentina
Source: PLoS Negl Trop Dis. 2014 May 22;8(5):e2894. doi: 10.1371/journal.pntd.0002894 (PMC4037315; doi:10.1371/journal.pntd.0002894)
Supplement: Table S4 — Multimodel assessment of factors associated with daily blood-feeding rates, human blood index, and human-feeding rates of T. infestans . Figueroa, spring 2003. (DOC) [file pntd.0002894.s006.doc]

**Table S4**. Multimodel assessment of factors associated with daily blood-feeding rates, human blood index, and human-feeding rates of *T. infestans*, Figueroa, spring 2003.

| **Daily feeding rates** | | | | | | | | | | | |
| --- | --- | --- | --- | --- | --- | --- | --- | --- | --- | --- | --- |
|  | | | Variables analyzed | | | | | | Model fit | | |
|  | Model | df | 1- Stage | 2-Chicken blood index | 3- Dog blood index | 4-Bug abundance | 5-Humans | 6-Maximum temperature | logLik | ∆AIC_i_^2^ | ω_i_ |
| 1 | 2 | 3 | – | X | – | – | – | – | -189.75 | 0.00 | 0.08 |
| 2 | 12 | 5 | X | X | – | – | – | – | -187.80 | 0.23 | 0.07 |
| 3 | 25 | 4 | – | X | – | – | X | – | -188.86 | 0.27 | 0.07 |
| 4 | 23 | 4 | – | X | X | – | - | – | -188.93 | 0.42 | 0.07 |
| 5 | 125 | 6 | X | X | – | – | X | – | -186.91 | 0.52 | 0.06 |
| 6 | 123 | 6 | X | X | X | – | – | – | -187.03 | 0.76 | 0.06 |
| 7 | 235 | 5 | – | X | X | – | X | – | -188.20 | 1.03 | 0.05 |
| 8 | 1235 | 7 | X | X | X | – | X | – | -186.30 | 1.39 | 0.04 |
| 9 | 24 | 4 | – | X | – | X | – | – | -189.46 | 1.49 | 0.04 |
| 10 | 245 | 5 | – | X | – | X | X | – | -188.59 | 1.80 | 0.03 |
| 11 | 124 | 6 | X | X | – | X | – | – | -187.63 | 1.97 | 0.03 |
| 12 | 26 | 4 | – | X | – | – | – | X | -189.71 | 1.98 | 0.03 |
| 13 | 126 | 6 | X | X | – | – | – | X | -187.68 | 2.06 | 0.03 |
| 14 | 234 | 5 | - | X | X | X | – | – | -188.78 | 2.18 | 0.03 |
| 15 | 236 | 5 | – | X | X | – | – | X | -188.81 | 2.25 | 0.03 |
| 16 | 1236 | 7 | X | X | X | – | – | X | -186.73 | 2.26 | 0.03 |
| 17 | 1245 | 7 | X | X | – | X | X | – | -186.75 | 2.29 | 0.03 |
| 18 | 256 | 5 | – | X | – | – | X | X | -188.86 | 2.34 | 0.03 |
| 19 | 1256 | 7 | X | X | – | – | X | X | -186.88 | 2.56 | 0.02 |
| 20 | 1234 | 7 | X | X | X | X | – | – | -186.97 | 2.73 | 0.02 |
| 21 | 2345 | 6 | – | X | X | X | X | – | -188.05 | 2.81 | 0.02 |
| 22 | 5 | 3 | – | – | – | – | X | – | -191.27 | 3.05 | 0.02 |
| 23 | 2356 | 6 | - | X | X | – | X | X | -188.18 | 3.06 | 0.02 |
| 24 | 12356 | 8 | X | X | X | – | X | X | -186.17 | 3.25 | 0.02 |
| 25 | 12345 | 8 | X | X | X | X | X | – | -186.23 | 3.37 | 0.02 |
| 26 | 15 | 5 | X | – | – | – | X | – | -189.40 | 3.43 | 0.01 |
| 27 | 246 | 5 | – | X | – | X | – | X | -189.42 | 3.46 | 0.01 |
| 28 | (Null) | 2 | – | – | – | – | – | – | -192.61 | 3.69 | 0.01 |
| 29 | 1246 | 7 | X | X | – | X | – | X | -187.51 | 3.82 | 0.01 |
| 30 | 2456 | 6 | – | X | – | X | X | X | -188.59 | 3.88 | 0.01 |
| 31 | 2346 | 6 | – | X | X | X | – | X | -188.65 | 4.00 | 0.01 |

Symbols: X (variable included in model), – (Variable not included in model)

∆AICc_i_= AICc_i_ - AICc_min_

ωi=exp(-1/2 ∆AICc_i_) / Σ exp (-1/2 ∆AICc_i_).

2 Lowest AICc= 385.57

| **Human blood index** | | | | | | | | | | | |
| --- | --- | --- | --- | --- | --- | --- | --- | --- | --- | --- | --- |
|  | | | Variables analyzed | | | | | | Model fit | | |
|  | Model | df | 1- Stage | 2-Chicken blood index | 3- Dog blood index | 4-Bug abundance | 5-Humans | 6-Maximum temperature | logLik | ∆AIC_i_^2^ | ω_i_ |
| 1 | 236 | 5 | – | X | X | – | – | X | -83.71 | 0.00 | 0.22 |
| 2 | 2346 | 6 | – | X | X | X | – | X | -82.76 | 0.18 | 0.20 |
| 3 | 23 | 4 | – | X | X | – | – | – | -85.06 | 0.63 | 0.16 |
| 4 | 234 | 5 | – | X | X | X | – | – | -84.37 | 1.32 | 0.11 |
| 5 | 2356 | 6 | – | X | X | – | X | X | -83.66 | 1.99 | 0.08 |
| 6 | 23456 | 7 | – | X | X | X | X | X | -82.74 | 2.24 | 0.07 |
| 7 | 235 | 5 | – | X | X | – | X | – | -85.05 | 2.68 | 0.06 |
| 8 | 2345 | 6 | – | X | X | X | X | – | -84.36 | 3.38 | 0.04 |
| 9 | 1236 | 7 | X | X | X | – | – | X | -83.50 | 3.76 | 0.03 |
| 10 | 123 | 6 | X | X | X | – | – | – | -84.64 | 3.94 | 0.03 |

Lowest AICc, 177.63

| **Human feeding-rates** | | | | | | | | | | | |
| --- | --- | --- | --- | --- | --- | --- | --- | --- | --- | --- | --- |
|  | | | Variables analyzed | | | | | | Model fit | | |
|  | Model | df | 1- Stage | 2-Chicken blood index | 3- Dog blood index | 4-Bug abundance | 5-Humans | 6-Maximum temperature | logLik | ∆AIC_i_^2^ | ω_i_ |
| 1 | 23 | 4 | – | X | X | – | – | – | -147.83 | 0.00 | 0.27 |
| 2 | 123 | 6 | X | X | X | – | – | – | -146.42 | 1.32 | 0.14 |
| 3 | 234 | 5 | – | X | X | X | – | – | -147.55 | 1.51 | 0.13 |
| 4 | 236 | 5 | – | X | X | – | – | X | -147.82 | 2.04 | 0.10 |
| 5 | 235 | 5 | – | X | X | – | X | – | -147.83 | 2.06 | 0.10 |
| 6 | 1234 | 7 | X | X | X | X | – | – | -146.25 | 3.07 | 0.06 |
| 7 | 1236 | 7 | X | X | X | – | – | X | -146.34 | 3.25 | 0.05 |
| 8 | 1235 | 7 | X | X | X | – | X | – | -146.41 | 3.39 | 0.05 |
| 9 | 2346 | 6 | – | X | X | X | – | X | -147.52 | 3.52 | 0.05 |
| 10 | 2345 | 6 | – | X | X | X | X | – | -147.55 | 3.58 | 0.05 |

Lowest AICc, 303.8
