# Supplementary material for: Domestic Animal Hosts Strongly Influence Human-Feeding Rates of the Chagas Disease Vector Triatoma infestans in Argentina
Source: PLoS Negl Trop Dis. 2014 May 22;8(5):e2894. doi: 10.1371/journal.pntd.0002894 (PMC4037315; doi:10.1371/journal.pntd.0002894)
Supplement: Text S1 — Sensitivity analysis of blood-feeding rates to changes in parameter estimates of the correction factor. (DOC) [file pntd.0002894.s007.doc]

Text S1 Sensitivity analysis of blood-feeding rates to changes in parameter estimates of the correction factor.

To see if our estimates of the blood-feeding rate are sensitive to a 10% perturbation (as an estimate of the uncertainty in the parameters of the formula c = 0.0533*t – 0.585), we used 10% upper and lower possible values of the slope 0.0533 by calculating 0.0533*1.1 and 0.0533*0.9, and similarly we used 10% upper and lower possible values of the intercept 0.585 by calculating 0.585*1.1 and 0.585*0.9. Then we estimated c(upper) = 0.0533*1.1*t - 0.585*0.9, and c(lower) = 0.0533*0.9*t - 0.585*1.1. Using these two formulas instead of the original one, we repeated the calculations for the temperature-adjusted overall proportion of domestic bugs with transparent urine per house, and obtained the following estimates for the median feeding interval (first-third quartiles) in 21 houses that had six or more bugs examined for transparent urine. Compared with the original (unperturbed) values for the median feeding interval (4.1 days, 2.4-7.0), these 10% perturbations of c yielded close values at 3.4 days (2.3-6.0) and 5.0 days (3.0-7.0).
